# Effectiveness of a group-based psychosocial program to prevent depression and anxiety in older people attending primary health care centres: a randomised controlled trial

**DOI:** 10.1186/s12877-019-1255-3

**Published:** 2019-08-29

**Authors:** Sandra Saldivia, Carolina Inostroza, Claudio Bustos, Paulina Rincón, Joseph Aslan, Vasily Bühring, Maryam Farhang, Michael King, Félix Cova

**Affiliations:** 1 Dpto. de Psiquiatria y Salud Mental Universidad de Concepcion, Concepcion, Chile; 2 Dpto. de Psicologia Universidad de Concepcion, Concepcion, Chile; 3 Division of Psychiatry, University College of London, London, England

**Keywords:** Promotion, Prevention, Mental health, Older people, The elderly, Depression, Anxiety

## Abstract

****Background**:**

Evidence about the effectiveness of psychosocial interventions to reduce the incidence of depression and anxiety and promote subjective well-being in older people is limited, particularly in Latin-American countries. This study thus aims to assess a program specifically designed to address this issue in persons aged 65 to 80 and attending primary health care centres.

**Method:**

Older people who use primary care centres are to be randomly assigned to the program or to a control group. Only independent users will be included; those having had a major depressive disorder or an anxiety disorder in the last 6 months will be excluded. The program is group based; it includes cognitive stimulation, expansion of social support networks and cognitive behaviour strategies. Depressive and anxiety symptoms and disorders, as well as psychological well-being, will be assessed using standardised instruments, once before implementing the program and later, after 18 and 36 weeks.

**Discussion:**

Primary care is a setting where interventions to improve mental health can be beneficial. Providing evidence-based programs that work with older people is a priority for public mental health.

**Trial registration:**

A protocol for this study has been registered prospectively at ISRCTN registry on 25 July 2018. Identifier: ISRCTN32235611.

## Background

Public policies and programs specifically designed to meet the needs of the growing elderly adult population are required. This is particularly relevant in the field of public health. Although 23% of the global burden of disease worldwide is attributable to disorders in people aged 60 and older, access to care and health coverage is particularly poor amongst them [1, 2].

Aging is a process that can affect the autonomy and quality of life [3]. Although psychiatric disorders are somewhat less common in older people than in younger adults, the prevalence rates vary between 8.4 and 26.4%, with significant impacts on both general health and the quality of life [4–6]. Worldwide, the prevalence of cognitive impairment in people aged 65 and older is approximately 10%, but this rate doubles by the age of 80 [4]. Similar rates are reported in Latin America, including Chile [4, 7, 8].

Promoting the psychological well-being and quality of life of the elderly and trying to prevent mental difficulties and disorders that most affect them are currently major challenges for public mental health [9]. These considerations are a fundamental part of an adult health care model, which is both comprehensive and addresses the close interrelationship between physical and mental health, particularly relevant for this population. In recent decades, the development of programs with these objectives has increased, particularly in high-income countries [10].

Given its magnitude and the impact on the quality of life of the elderly and their families, a central concern should be the prevention of common mental disorders: depression and anxiety. There is growing evidence of the effectiveness of psychosocial interventions aimed at preventing the occurrence of depression and/or anxiety in older adults and/or promoting their subjective well-being [10–13]. It is not unclear whether greater effectiveness is observable in focused or universal programs, although it is thought to be more favourable in the former [10, 14]. However, even when effectiveness appears greater in targeted interventions, universal programs can have a wider impact on the population if they allow access to more people with a moderate risk than merely those with high risk [15]. Lee, Franchetti [16] summarised the findings of 5 studies of the indicated prevention in the elderly population, with subthreshold depressive symptoms. They reported that positive results were evident for several psychotherapeutic strategies, either used alone or accompanied by other components. Among the therapeutic strategies used were the following: review of one’s life, behavioural activation, problem solving and broader cognitive-behavioural strategies [17–20]. Furthermore, multi-stage interventions have proved both useful and cost-effective [21].

Despite these promising results, there exists no evidence in Latin America regarding the effectiveness of preventive or promotional programs in mental health in older adults. This study thus purports to design, implement and evaluate a multi-component intervention aimed at preventing depressive disorders in people aged 65 to 80, who consult primary care centres in Chile, where primary care services are effectively the gateway to the health system. Over 70% of the population access this system; it is where the largest proportion of older adults go for medical attention [22]. Given the close interrelation between depression and anxiety, effects are consequently expected in relation to the prevention of anxiety and the promotion of psychological well-being, specifically, with an increase in satisfaction with life. The program was designed to be short, to facilitate the eventual scaling up in primary care, but bearing in mind that excessively brief programs do not always show positive results [10]. It is a universal program, aimed at the self-reliant older population, who use primary care centres and have not encountered a depressive or anxious disorder in the past year. The intervention is group oriented, consisting of 9 face-to-face sessions and telephone follow-ups. It has been designed considering components shown to have most contributed to the effectiveness of such programs in other settings, including cognitive stimulation, the extension of social support networks and cognitive behaviour strategies, such as relaxation training, behavioural activation and problem solving [16].

It is expected that the intervention will be effective in reducing symptoms of depression and anxiety in some older participants, increasing psychological well-being. Follow-up evaluations, at 18 and 36 weeks, will permit a comparison between the trial arms of the levels of depressive and anxious symptoms, measured as continuous variables. Furthermore, the incidence of depressive and anxious disorders in both groups at the 36-week follow-up point will be compared. Note, we are aware that demonstrating the direct effects in reducing disorders’ incidence in universal programs requires very large samples and long follow-ups [23]. Thus, we will regard any impact on depressive and anxious symptoms and psychological well-being as important proximal risk factors in the development of the disorders themselves.

## Methods/design

**Fig. 1:**
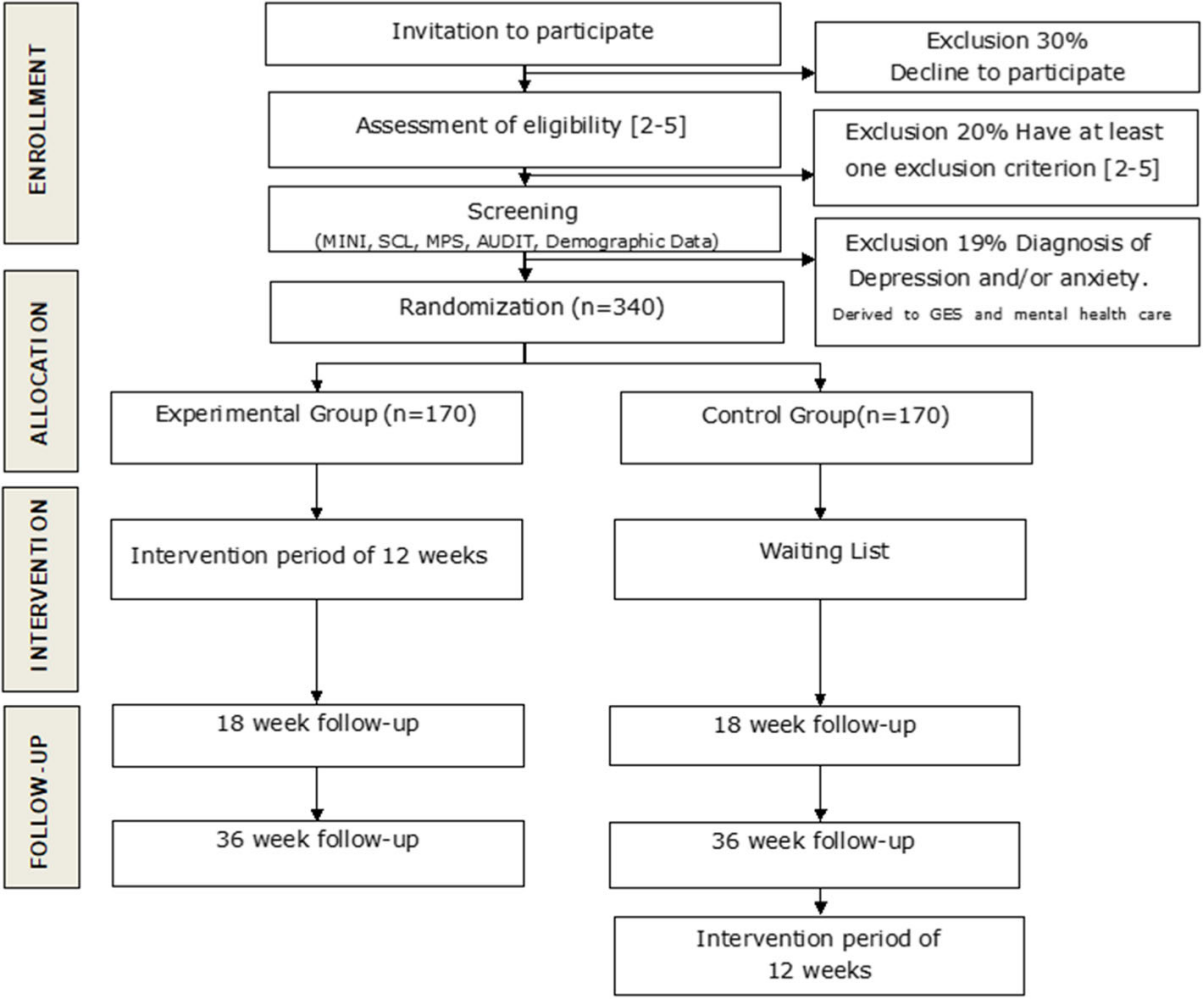
Participant flow through the study

This is a randomised clinical trial with block randomisation conducted in 15 primary health care centres. This study protocol has followed the SPIRIT guidelines [24]. The trial has been registered with the protocol number ISRCTN32235611. An overview is presented in Fig. 1.

The inclusion criteria for participants will be self-dependent men and women between 65 and 80, without major depressive episodes, generalised anxiety disorder or panic disorder, attending the primary care centres. Exclusion criteria are the presence of a severe mental disorder (psychosis, bipolar disorder), mental disability or dementia or a disability preventing communication. The assessment of these criteria is to be made from the health records by the health personnel using a standardised instrument (EMPAM) [25]. An EMPAM score greater than 42 indicates the person is self-dependent and able to provide consent. Potential participants will be invited by a research assistant during a home visit. Those who agree to participate and provide written informed consent will receive the initial evaluation. During the initial evaluation, participants meeting the diagnostic criteria for major depressive disorder, generalised anxiety disorder or panic disorder will be excluded from the study (and if not already receiving treatment, will be referred to the mental health unit of the respective health centre).

Participants meeting the inclusion criteria and agreeing to participate will be randomised to the experimental or waiting list control group, which will receive the intervention after the last assessment. To ensure similar numbers of participants per centre and a balance of both sex and age in each trial arm, block randomisation will be carried out. Each block is made comprised a combination of the health centre, a specific age group (65–74/70–74/75 and above) and gender (Male/Female). Allocation sequence was made with computer-generated random numbers. Randomisation will be conducted once the initial evaluation is done for all participants in each health centre by an independent statistician.

To estimate the size of the sample, the meta-analysis of Forsman, Nordmyr [10], which reported a reduction in depressive symptoms of standardised mean difference (SMD) = − 0.32 in universal prevention interventions in older adults, was taken as a reference. It was estimated that a sample of 154 participants per arm at the time of the final evaluation would be sufficient to detect significant differences using a conservative test (t-test for independent samples of differences in the post-test), with a power of 80% and a 5% level of significance. Taking into account expected attrition, we will need to recruit 170 participants in each arm. This will imply conducting approximately 15 intervention groups in the participating health centres, each of which will contain 10 to 12 people.

To evaluate the possible design effect on sample size, the method for sample size calculation in multi-centre clinical trials by Harden and Friede [26] was used. Setting a ICC = 0.1, number of centres = 15 and an estimated block size of b = 4, the total sample size needed was 307, so no important effect on sample size could be attributed to design.

Participants will be evaluated in a home visit during recruitment and later, at 18 and 36 weeks after the intervention ends. An overview of the data collection is presented in Table 1. All measures will be carried out at each assessment point; only the presence of depressive disorder, generalised anxiety disorder and panic disorder will be measured at the baseline, to confirm exclusion criteria, and 36 weeks later, to evaluate the presence of a diagnosis. The initial evaluations and the 2 post-intervention evaluations will be carried out in participants’ homes by trained interviewers, blind to trial arm allocation.

**Table 1:** Participants Timeline

	STUDY PERIOD					
	Enrolment	Allocation	Post-allocation			
TIMEPOINT**	-t_*1*_	0	t_*1*_ *baseline*	*t*_*2*_ *intervention*	*t*_*3*_	*t*_*4*_
ENROLMENT:						
Eligibility screen	X					
Informed consent	X					
Identification of “key-persons” in PHC and meetings together with representatives from the research group		X				
Allocation		X				
INTERVENTIONS:						
Psychosocial intervention				X		
ASSESSMENTS:						
EMPAM	X					
CIDI 2.1			X			X
MINI			X			X
PHQ-9			X		X	X
SCL-90-R			X		X	X
MSPSS			X		X	X
AUDIT			X		X	X
PHI			X		X	X
ULS-3			X		X	X
Sociodemographic questionnaire			X		X	X

*t*_*1*_ *baseline, t*_*2*_ *intervention period, t*_*3*_ *18 week follow up, t*_*4*_ *36 week follow up*

### Measures

The following measuring instruments will be used:

#### EMPAM Chile: preventive medicine examination of the elderly [25]

This annual preventive exam evaluates the integral health, functionality and main risk factors of the elderly. This standardised instrument is part of the usual control of older adults in primary health centres in Chile. Scores of 42 points or less indicate the person is in risk of dependence [27].

#### CIDI 2.1

Structured diagnostic interview developed by the World Health Organisation (WHO) [28]. The CIDI provides diagnoses according to the criteria in DSM-IV and ICD-10, for the preceding 12 months and/or 30 days. It has been widely used in epidemiological studies in general populations and primary care. It has adequate inter-rater and test-retest reliability, and evidence of validity according to multiple studies [29]. Only section E (on major depressive disorder), validated in Chile [30], will be used.

#### MINI international neuropsychiatry interview (MINI) [31, 32]

A brief structured diagnostic interview assessing psychiatric disorders according to ICD-10 and DSM-IV criteria. The MINI has high reliability and validity; it requires brief administration [31]. Version 5.0 in Spanish has been validated by Ferrando, Franco-Alfonso [32] and used widely in Chile in studies of depression [33, 34]. In this study, the section referring to generalised anxiety and panic disorder will be applied.

#### Patient health questionnaire (PHQ-9) [35, 36]

The PHQ-9 is a nine-item self-report measure evaluating the presence of depressive symptoms over the preceding 2 weeks based on DSM-IV criteria for major depressive episode. The response scale is a Likert-type frequency scale of 4 points: 0 = never; 1 = on several days; 2 = for over half of the days; 3 = almost every day. The instrument has been translated into Spanish and validated in Chile, confirming its reliability (Cronbach’s alpha is 0.83) and factorial structure [35].

#### Symptom check list (SCL-90-R) [37, 38]

This 90-item self-administered multidimensional questionnaire is designed to assess the range of psychopathological problems in medical or psychiatric patients or in the general population. Internal consistency if its sub-scales varies from 0.78 to 0.90. The anxiety subscale will be used. In its use in Chile, it has shown adequate psychometric properties [38].

#### Multidimensional scale of perceived social support (MSPSS) [39, 40]

This instrument is composed of 12 items on perceived social support in 3 areas: family, friends and significant others. Its response scale corresponds to a Likert-type scale ranging from 1 = strongly disagree to 7 = strongly agree. The instrument has been translated into Spanish; its use has been validated in the Chilean population [40]. As a result of this process, researchers adapted the response scale to a Likert-type frequency scale of 4 points: 1 = almost never; 2 = sometimes; 3 = frequently and 4 = always or almost always. Its adaptation for use in the Chilean population showed good evidence of reliability (Cronbach’s alpha is 0, 86) and a clear factorial structure in older adults.

#### Alcohol use disorders identification test (AUDIT) [41, 42]

Self-applied questionnaire of 10 items. The first 8 assess alcohol consumption, risk of dependence and negative consequences in the last 12 months, while the last 2 assess risk over the participant’s lifetime. In Chile, the instrument has a sensitivity of 80% and a specificity of 89% for a score of 9 and above for both harmful consumption and risk of dependence [42].

#### The Pemberton happiness index [43, 44]

Self-applied questionnaire including 2 components of well-being. The first, with 11 items, assesses the overall well-being and its 4 domains: general well-being, psychological well-being (eudaimonia), subjective well-being (hedonia) and social welfare. The second, of 10 items, assesses well-being experienced as a result of positive and negative experiences from the previous day. In the original validation study involving 7 languages (including Spanish and Mexican populations), the internal consistency was 0.89 and the inter-item correlations varied between 0.31 and 0.56. The instrument has also been validated using samples from Chile, Cuba and Uruguay, showing adequate psychometric properties [44].

#### UCLA loneliness scale version 3 (ULS3) [45, 46]

This self-report measure evaluates the subjective experience of loneliness and social isolation. It consists of 20 items, 11 negatives and 9 positives, where each question begins with "How often do you feel … " with a Likert format of 4 responses: 1 = never; 2 = rarely; 3 = sometimes and 4 = many times. The instrument has been validated in Spain with samples of older people and shows high internal reliability (Cronbach’s alpha 0.95) and construct validity [46].

#### Sociodemographic background questionnaire

This will include sex and age, family structure and the quality of the couple’s relationship, based on 2 questions from the PREDICT Chile questionnaire [47]; furthermore, it will include information on possible stressful events in the family in the last year, based on the List of Threatening Experiences (LTE) [48], in its Spanish version [49].

### Analysis

Data quality will be handled using double data entry. All the main analysis will be made using intention to treat principle. Missing data will be handled using multiple imputation. The analysis of the main outcome, the reduction of depressive symptoms, will be made using a general linear model of mixed effects, with adjustment for potential confounding variables. Allocation to each trial arm, time and the interaction effect group X time, on which the efficacy of the treatment is tested, will be considered as the fixed effects. Confounding variables which are important in the outcome of depressive symptoms will be considered in the analysis as fixed effects – namely, social support, individual risk factors, physical illness and alcohol consumption. The primary care centre will be entered as a random effect. This analysis will be repeated for both anxiety symptoms and well-being.

The analysis of secondary outcomes (presence of major depressive disorder, generalised anxiety and panic disorder) will be performed with a generalised linear model with a logit link, corresponding to a logistic regression with mixed effects, and analogous to the linear model used in the symptoms’ analysis.

### Intervention program

A program, Active Life, was designed, with a general objective to prevent depression and anxiety and improve the quality of life of older people using primary health care. Its specific objectives are as follows: to enhance the capacity to enjoy life in older adults, maintain physical health by encouraging physical activity and relaxation, maintain cognitive functioning and apply problem-solving strategies for common difficulties.

Based on available evidence on prevention programs for depressive and/or anxiety symptoms in older adults [10, 14, 16], the intervention program includes the psychoeducation about self-care for each participant to identify ways in which to modify their lifestyle, with a focus on the following: [1] physical health, cognitive activity, emotional, social and spiritual life, and sleep hygiene, and how to implement the same; [2] training in relaxation through mindfulness, with practice in each session and the delivery of material for practice at home; [3] behavioural activation in terms of a program of enjoyable activities encouraging activities of a recreational, occupational and/or social nature and valued positively by the participant, which to a feasible extent increases social participation, making use of available resources in the community; [4] problem solving training for the daily difficulties of older age and the management of anxious and depressive symptoms; [5] cognitive stimulation, through games designed for this purpose (baccalaureate, sudoku, bingos and memorise) and [6] empowerment of the changes through an inter-session telephone follow-up and after the close of the face-to-face program. Each session is designed to be delivered in a group format, since this favours contact, support and social integration, as well as the sharing and containment of subjects personal to the participants (recent emotional losses, changes of role, diminution of their abilities and the appearance or aggravation of health problems). The intervention period will last for 12 weeks, with 9 face-to-face sessions of 2 h each week, and 2 telephone follow-ups- the first in the break week between session 8 and 9; the second 2 weeks after the last face-to-face session.

The group sessions are conducted by a facilitator (psychologist) and co-facilitator (health professional), both with 30 h of training in the program. Facilitators will receive telephone and face-to-face supervision every week, where spontaneous or adverse events will be analysed. The intervention includes the delivery of material supporting the participants in undertaking plans made during sessions (brochures, diaries), complementing the playful work of cognitive training (prizes for the winners of games such as bingo or sudoku) and information on social resources available for older adults and how to contact the same. As strategies to improve adherence, the systematic monitoring of each participant will be considered through weekly telephone calls and support with payment of transportation for those requiring it.

## Discussion

This clinical trial uses block randomisation to evaluate a multi-component psychosocial intervention aimed at reducing symptoms of depression and anxiety and increasing the well-being in older adults visiting primary care centres in Chile. The intervention has been designed specifically for evaluation in this study.

There is emerging evidence of the effectiveness of psychosocial interventions to prevent depression and/or anxiety in older adults and/or promote their well-being, although this remains inconclusive. There exist no other ongoing evaluations of this approach in Latin America, despite its obvious relevance. An ageing population is already a reality in these countries. In Chile, adults over 65 comprise of 11.4% of the population; this group is projected to increase to over 15% by 2030 [50].

This program will contribute to the reduction of depression and anxiety and the promotion of psychological well-being in older adults. It has been developed based on strategies that showed the greatest effectiveness in other research; given its delivery in primary care centres, it has the potential to improve public mental health. The program has been carefully designed and documented and will be subjected to a rigorous evaluation for effectiveness. The results could help future research evaluate the potential of the program to be part of a global strategy geared towards improving the quality of life of older people in Latin America.

The results will be published in scientific journals, presented at conferences and made available in suitable form to the general public.

## Data Availability

Any person wishing to access the full dataset must submit a formal request to the Principal Investigator for approval. A database with the individual global score in each questionnaire will be available in an OSF project, identifying in each case the health centre, the intervention or control group, the sex and age of participants.

## Abbreviations

DSM-IV: Diagnostic and Statistical Manual of Mental Disorders IV edition
EMPAM: Preventive Medicine Examination in the Elderly
ICD-10: International Classification of Diseases 10th Edition
SMD: Standardised Mean Difference
